# Subscapularis Transthoracic Versus Posterolateral Approaches in the Surgical Management of Upper Thoracic Tuberculosis: A Prospective, Randomized Controlled Study

**DOI:** 10.1097/MD.0000000000001900

**Published:** 2015-10-30

**Authors:** Bin Lin, Ji-Sheng Shi, Hai-Shen Zhang, Chao Xue, Bi Zhang, Zhi-Min Guo

**Affiliations:** From the Department of Orthopaedics (BL, J-SS, H-SZ, CX, Z Z-MG), the 175th Hospital of PLA, Orthopaedics Center of PLA, Southeast Hospital of Xiamen University, Zhangzhou, Fujian, People's Republic of China; and Department of Orthopaedics (BZ), Ganzhou People's Hospital, Ganzhou, Jiangxi, People's Republic of China.

## Abstract

The objective of the present study was to evaluate the clinical, radiological, and functional outcomes of a subscapularis transthoracic surgical approach and a posterolateral surgical approach with debridement, bone graft fusion, and internal fixation for the treatment of upper thoracic tuberculosis.

There is currently debate over the best surgical approach for the treatment of upper thoracic tuberculosis. Traditionally, the subscapularis transthoracic approach has been preferred; however, the posterolateral approach has gained popularity in the past few years.

A prospective, consecutive cohort of 43 upper thoracic tuberculosis patients with a mean age of 39 years (range: 20–52 years) was followed up for a minimum of 12 months (range: 12–60 months). Patients were randomly divided into 2 groups. Group A (n = 21) was treated by the subscapularis transthoracic approach and group B (n = 22) was treated by the posterolateral approach. All cases were evaluated for clinical, radiological, and functional outcomes. Intraoperative blood loss, operative duration, intraoperative and postoperative complications, hospital stay, the cure rate, fusion time, and the Frankel scale were used for clinical and functional evaluation, whereas the kyphosis angle was used for radiological evaluation.

Grafted bones were fused by 10 months in all cases. There was no statistically significant difference between groups before surgery in terms of gender, age, segmental tuberculosis, erythrocyte sedimentation rate (ESR), Frankel scale, or Cobb's angle (*P* > 0.05). The average operative duration for Group B was lower than that of Group A. There were no significant differences in intraoperative blood loss, intraoperative and postoperative complications, hospital stay, grafted bone fusion time, or cure rate between groups (*P* > 0.05). The Cobb's angle correction rate for group B (68.5%) was significantly better than that of group A (30.9%). The neurological score showed significant postoperative improvement in both groups, with no significant difference between the groups.

The subscapularis transthoracic approach and the posterolateral approach with debridement, bone graft fusion, and internal fixation are both sufficient and satisfactory for the surgical treatment of upper thoracic tuberculosis. However, the posterolateral approach is superior to the subscapularis transthoracic approach in terms of surgical trauma, operative time, and kyphosis correction.

## INTRODUCTION

Spinal tuberculosis is prevalent in many developing countries. It is the most dangerous form of bone and joint tuberculosis due to serious ensuing complications, such as severe neurological deficits, kyphotic deformities, and paraplegia.^1–3^ Because of the spine's complex anatomy and limited accessibility, various upper thoracic surgical approaches have been explored over the past several decades,^4–6^ with continual debate over the best approach for the treatment of spinal tuberculosis.^7^ In 1957, Cauchoix and Binet^8^ pioneered the full sternotomy approach for the treatment of upper thoracic tuberculosis. Although employed for decades, this approach resulted in a higher incidence of postoperative infection, increased operation trauma, and longer recovery time. In 1982, Standefer et al^9^ reported a method for the treatment of a cervical thoracic tumor, which involved the splitting of the sternum and removal of the inner part of the clavicle. This method, however, can result in shoulder girdle weakness, clavicular nonunion, sternal nonunion, and retrosternal soft tissue injury. In 1987, Turner and Webb^10^ first presented the subscapularis transthoracic approach, which became a widely adopted fixation method for the treatment of upper thoracic tuberculosis due to its improved safety, convenience, and reliability. Unfortunately, this approach has many disadvantages, including larger trauma, complications that limit shoulder function, and lower kyphosis correction ability. Thus, controversy remains regarding the best anterior approach and posterior instrumentation for lesion removal, reliable fixation, and reduced surgical trauma.^11^

The limitations of these approaches have driven some surgeons to advocate for a posterolateral approach to reveal upper thoracic. In 1894, Menard presented a posterolateral approach involving the removal of a section of the ribs for the surgical treatment of upper thoracic tuberculosis. This approach has now been employed for decades, with reports indicating that it is relatively safe and has satisfactory clinical effectiveness.^12^ In recent years, following the development of posterior pedicle screw segmental instrumentation and the improvement of operation skills, this approach has become more widely accepted for the treatment of upper thoracic tuberculosis.

The objective of this study was to compare the clinical, radiological, and functional outcomes of the subscapularis transthoracic approach and the posterolateral approach with debridement, bone graft fusion, and internal fixation for the surgical treatment of upper thoracic tuberculosis. The 2 approaches were evaluated in terms of intraoperative blood loss, operation duration, bony fusion, intraoperative and postoperative complications, hospital stay duration, cure rate, Frankel scale, and kyphosis angle.

## MATERIALS AND METHODS

### Study Population

Forty-three consecutive patients with upper thoracic tuberculosis were evaluated in this prospective study. These patients were randomly divided into 2 groups according to their sequence of admission using a table of random numbers, with 21 patients in group A and 22 in group B. Even group (group A) was treated using the subscapularis transthoracic approach and odd group (group B) was treated using the posterolateral approach. The indications for surgery included patients with upper thoracic tuberculosis (T1 ∼ T4) for whom anti-tuberculosis drugs were ineffective and who may have also suffered from greater sequestrum formation, sinus formation, larger abscesses, serious vertebral collapse, progressive kyphosis, and neurological deficits. Surgical contraindications were patients with liver, heart, and kidney dysfunction; highly infectious tuberculosis; or serious underlying lung disease. The exclusion criteria included patients younger than 18 years with a history of upper thoracic surgery and incomplete follow-up information, and patients with a distant stream abscess that would require anterior surgery or multiple incisions.

All patients in the study provided their written informed consent. This study was approved by the institutional review board and the ethics committee of the 175th Hospital of PLA.

### Preoperative Examination and Preparation

Standard laboratory tests were performed for all patients and included erythrocyte sedimentation rate (ESR), blood chemistry profiling, white and red blood cell count, and a Mantoux tuberculin skin test. An electrocardiogram, a radiograph of the chest, magnetic resonance imaging (MRI), and computerized tomography (CT) were also performed for all patients. Narrowing of the intervertebral space and different degrees of vertebral collapse were observed during X-ray examination for every patient. For every case, different degrees of sequestration were observed during CT examination, and varying degrees of vertebral body destruction were observed during MRI examination. Thirty-seven cases had varying degrees of spinal cord compression, and 35 cases exhibited paravertebral abscess. No patients had sinus formation.

After the preliminary diagnosis of upper thoracic tuberculosis, all patients received standard anti-tuberculosis chemotherapy, including oral administration of pyrazinamide (0.5 g per day), isoniazid (0.3 g per day), rifampin (0.45 g per day), and ethambutol (0.75 g per day) for 2 to 4 weeks. ESR was tested on alternate weeks, and surgery was performed when the changes in ESR did not exceed 10 mm/h and did not display an increasing trend.

### Surgical Technique

For all patients in group A, subscapularis transthoracic surgery was performed under general anesthesia. First, patients were placed in the lateral position, and a midline incision was made from the C7 or T1 spinous process to the junction of the anterior axillary line and 4,5 intercostal via the medial of spealbone. Then, the muscle and fascia were incised, and the loose connective tissue was separated between the scapula and the chest wall. If necessary, surgeons exposed the lesions on the vertebra by resecting the ribs (specifically, rib 3, 4, or 5) and by using curettes to debride the affected vertebral body, infected tissue, pus, granulation tissue, sequestrum, and disc necrotic tissue. Third, an intervertebral bone autograft from the iliac crest and Ventrofix fixation system (AO, Grisons, Switzerland) was used. Streptomycin (2 g) was sprayed onto the operation site, and a local drainage tube was inserted before the incision was closed.

In group B, all surgical operations were performed with the patients placed in a prone position under controlled general anesthesia with endotracheal intubation. All 22 patients were treated with a posterolateral surgical approach with debridement, bone graft fusion, and internal fixation. Through a midline incision, the posterior spinal construction was exposed, including the spinous processes, lamina, facet joints, and transverse processes. With C-arm fluoroscopy assistance, pedicle screws or hooks were placed at least 2 levels superior and inferior to the level of decompression. The spine was stabilized using a temporary fixed rod to avoid spinal cord injury caused by debridement and decompression. To drain prevertebral abscesses and to expose diseased vertebral bodies, surgeons performed a costotransversectomy and removed the articular process, pedicle, and transverse process on the more severely affected side of the lesion segment. Spinal tuberculosis was revealed by the separation of the posterior longitudinal ligament and dural sac. The posterior longitudinal ligament was then incised, and spatulas of various sizes and angles were used from a posterolateral approach to remove lesions, including sequestra, abscesses, and granulation tissue. An appropriately sized iliac bone was removed from the posterior superior iliac spine and was implanted in the interbody. If necessary, the intercostal nerve was cut off. Streptomycin was applied as for group A. Eight patients with obvious kyphosis were corrected by applying appropriate pressure. Drainage and incisional sutures were performed postoperatively.

### Postoperative Care

Blood pressure, respiration, pulse, amount of drainage, and sense and motor responses of the lower extremities were monitored after the operation. The drainage tube was removed when the amount of drainage was less than 10 mL per day. Mannitol was used for the treatment of edema for 3 to 5 days, while neurotrophic therapy was performed for patients with neurological dysfunction. All patients received antituberculosis chemotherapy with the 4 drugs mentioned above for 12 to 18 months. Postoperative and preoperative anti-tuberculosis treatment programs were the same. All patients were immobilized in a rigid external orthosis for 3 to 4 months after surgery. Follow-up examinations were performed for all patients at 1, 3, 6, 9, and 12 months after surgery, and subsequent follow-ups were conducted at 12-month intervals. At each follow-up evaluation, liver and kidney function, routine blood test, C-reactive protein, and ESR were monitored carefully to assess the presence of active disease. Routine lateral and anteroposterior X-ray, CT, and MRI imaging was performed to evaluate the extent of decompression, fusion status, functional nerve recovery, and Cobb's angle of kyphosis.

### Evaluation Standards

For assessment of the correction rate, the kyphosis correction rate was defined as: (preoperative Cobb's angle – postoperative Cobb's angle)/preoperative Cobb's angle × 100%. Bone grafting fusion was assessed using the radiological criteria of Bridwell et al.^13^ The criteria for clinical cure of spinal tuberculosis included good general condition; absence of fever; normal appetite; absence of local pain; X-ray imaging of vertebral healing with a clear outline of the lesion; uniform MRI signal among the vertebral lesion, the surrounding tissue, and the normal vertebrae; and consistent normal ESR results. Patients were considered to have recurrence of spinal tuberculosis if the spinal tuberculosis was cured after surgery, but the primary lesion and corresponding symptoms reappeared after 1 year.

### Statistical Analysis

All statistical analyses were performed using SPSS 21.0 software. Classified variables and continuous variables were expressed by frequency and means ± standard deviation, respectively. A Wilcoxon rank sum test and *t* test were conducted for comparisons between groups A and B, and a *P* value <0.05 was considered statistically significant.

## RESULTS

From January 2008 to December 2013, we treated a total of 43 patients with upper thoracic tuberculosis by either a subscapularis transthoracic approach or a posterolateral approach. Patients in the 2 study groups were comparable (*P* > 0.05) with respect to their gender, age, segmental tuberculosis, ESR, Frankel scale, and Cobb's angle (Table 1). All patients were followed up for a minimum of 12 months (range: 12–60 months; average: 32.0 ± 12.9 months in group A vs 33.2 ± 13.0 mo in group B, *P* > 0.05), with no cases lost during the follow-up period. The follow-up time was no statistical significance between the 2 groups (*t* = −0.311, *P* = 0.757).

**TABLE 1:**
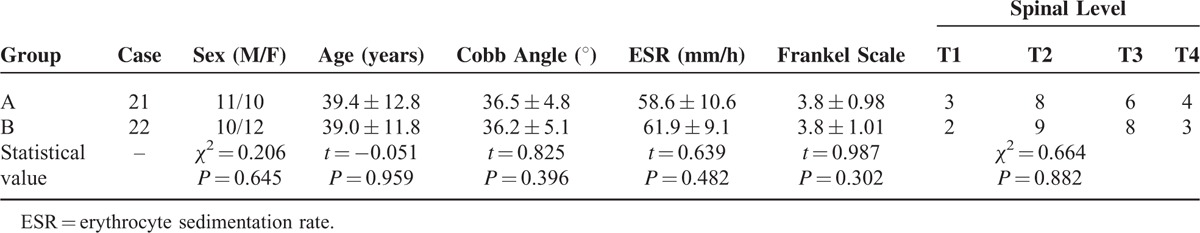
Demographic Characteristics (Mean ± Standard Deviation)

The surgical time was significantly shorter in group B than in group A (*P* < 0.05), while the differences in blood loss, length of hospital stay, grafted bone fusion time, and cure rate were not statistically significant (Table 2). In group A, 20 cases were cured (cure rate of 95.23%), while in group B, 21 cases were cured (cure rate of 90.91%) (*P* = 0.306). For 3 cases that demonstrated recurrence, antituberculosis treatment was administered and surgical treatment with a posterolateral approach was performed after the ESR was stable.

**TABLE 2:**
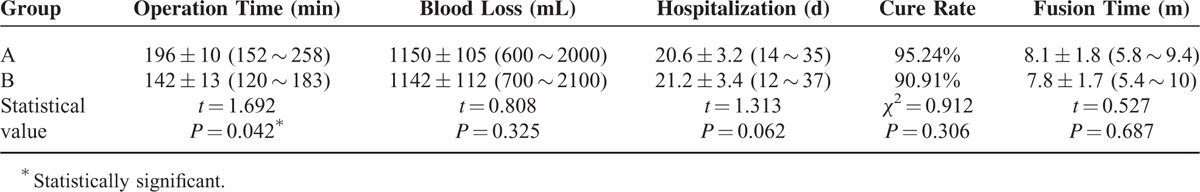
Comparison of Operation Time, Blood Loss, Time Spent in the Hospital, Grafted Bones Fusion Time and Cure Rate (Mean ± Standard Deviation)

**FIGURE 1:**
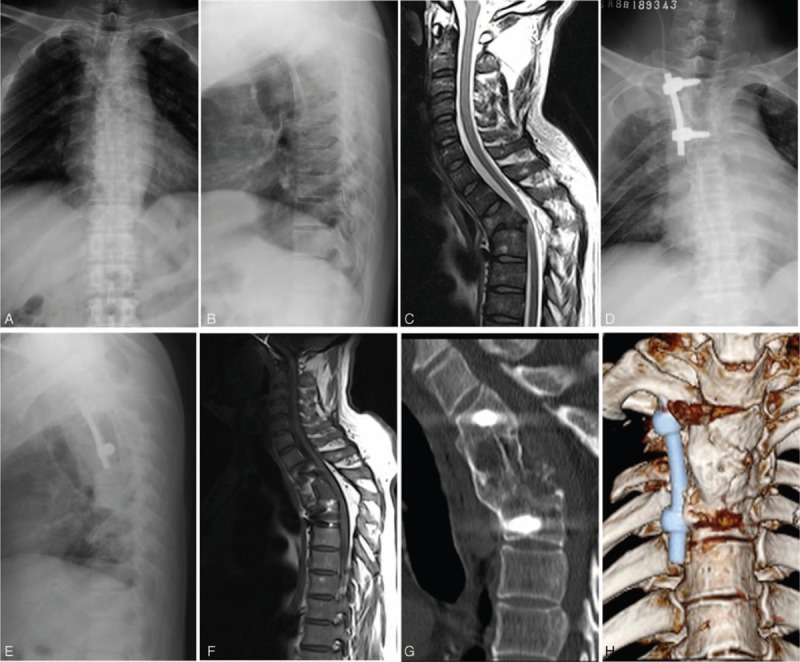
A 45-year-old man with spinal tuberculosis at T2 was treated by the subscapularis transthoracic approach. Preoperative anteroposterior and lateral X-ray imaging and sagittal MRI (A–C) showed T2 vertebral body destruction, collapse, disappearance of the intervertebral space, cord compression, and a local kyphosis angle of 44°. Postoperative thoracic lateral X-ray (D, E) of the same patient revealed good fixation location and a kyphosis angle of 37°. The 12-month follow-up examination showed satisfactory spinal decompression and no significant loss of kyphosis angle (36°) (F). The CT image and 3D reconstruction (G, H) showed that the bone graft had healed, with no lesion recurrence at T2.

**FIGURE 2:**
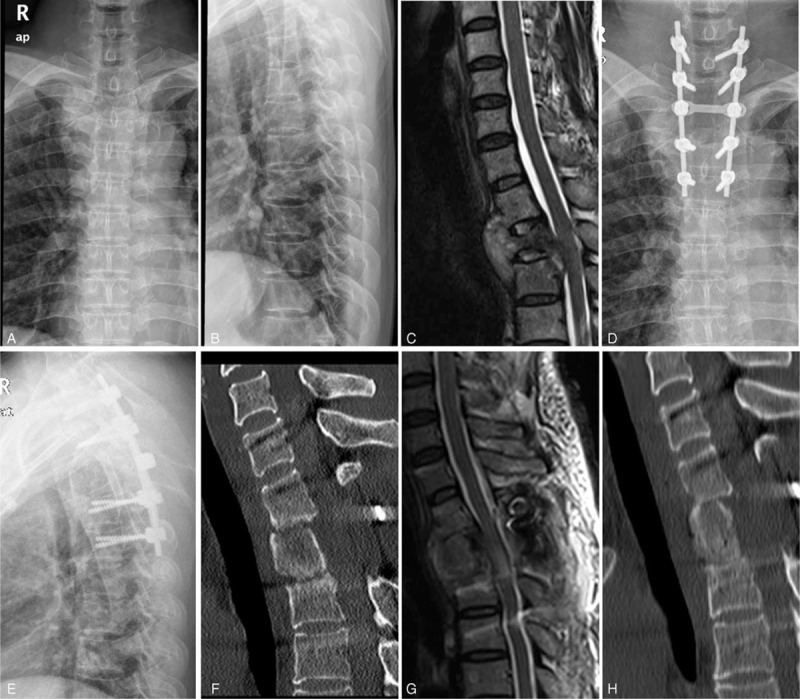
Preoperative anteroposterior and lateral X-ray of a 39-year-old female with tuberculosis of T2, who presented with a local kyphosis angle of 20° (A, B). Sagittal T2 WI (C) images showed active tuberculosis with abscess formation, vertebral destruction, and cord compression. This patient was treated by the posterolateral approach with pedicular screw-rod fixation. Postoperative X-rays (D, E) of the same patient showed good decompression and kyphosis correction, with a kyphosis angle of 9°. Postoperative sagittal thoracic CT imaging showed good positioning of the grafting iliac bone (F). Postoperative sagittal T2 WI (G) images showed no significant residual lesions around T2 and complete spinal cord decompression. At the 8-month follow-up examination, a sagittal thoracic CT revealed solid bony fusion, no loss of kyphosis correction, and no obvious recurring lesions around T2 (H).

Cobb's angles were measured as described by Carman et al.^14^ Both groups exhibited a statistically significant decrease in local kyphosis in the upper thoracic spine (T1–T4) after surgery (*P* < 0.05). Group B exhibited a greater kyphosis correction (Figures 1 and 2) and a higher correction rate (Table 3) than group A.

**TABLE 3:**
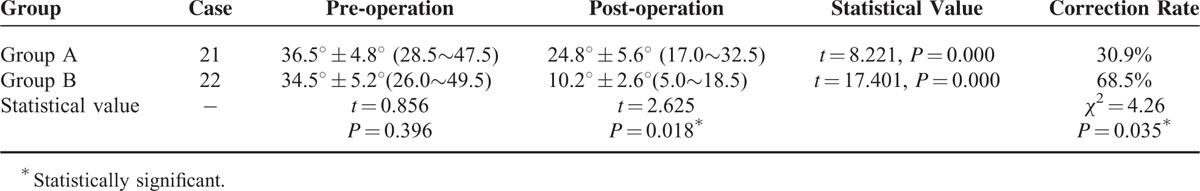
Preoperative and Postoperative Cobb Angle and Correction Rate (Mean ± Standard Deviation)

At the 12-month follow-up examination, each group demonstrated an improved postoperative neurological score compared with preoperative levels (*P* < 0.05), as evaluated by the Frankel scale (in group A: *Z* = −4.850, *P* = 0.000; in group B: *Z* = −5.494, *P* = 0.000). However, the difference between the 2 groups was not statistically significant (preoperative: *Z* = −0.808, *P* = 0.419; postoperative: *Z* = −0.033, *P* = 0.973, Table 4).

**TABLE 4:**
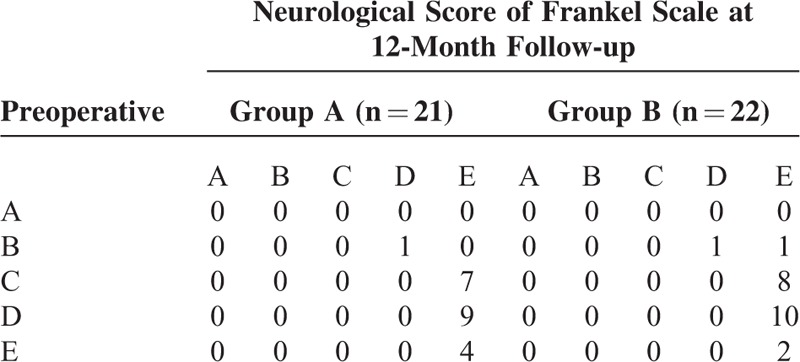
Neurological Score of Frankel Scale of Pre-operation and Post-operation (12-Month Follow-up)

In group A, 2 cases with postoperative shoulder abduction movement disorder (abduction angles: 0 ∼ 90° and 0 ∼ 110°) were treated by rehabilitation therapy, and movement was recovered at the 6-month follow-up examination. There were no other complications, such as leakage of cerebrospinal fluid or nerve injury aggravation. The complication rate for group A was 9.5%. In group B, 2 patients exhibited leakage of cerebrospinal fluid; thus, the usage of drainage pipes was prolonged. There were no other complications, such as wound infection, shoulder movement disorder, or nerve injury aggravation. The complication rate for group B was 9.1%. The incidence of complications between the 2 groups was not statistically different (*X*^2^ = 1.862, *P* = 0.125).

## DISCUSSION

There has been considerable debate over the best surgical approach for the treatment of upper thoracic tuberculosis. Surgical treatment of this disease must combine expertise in cervical lordosis and thoracic kyphosis with knowledge of the complex anatomy and important blood vessels, nerves, lymphatic vessels, and other structures within this anatomical region. There have been several anterior approaches reported in the literature, including a presternal approach^8^ and an improved presternal approach with partial clavicle resection,^9^ which has been used by many surgeons for upper thoracic tuberculosis lesions.^3,15^ In one report,^16^ 140 patients who underwent anterior approach initially exhibited a 51.0% correction of kyphosis; however, the rate of correction dropped to 7.5% after 2 years. Cui et al^17^ reported that in 181 patients with spinal tuberculosis, the degree of kyphosis was corrected by a mean of 11.5° in the anterior group and 12.6° in the posterior group (*P* < 0.01). The kyphosis correction decreased by 6.8° in the anterior group and 6.1° in the posterior group when measured at the last follow-up examination (*P* < 0.01).

A subscapularis transthoracic approach for the treatment of thoracic tuberculosis has many advantages, such as clear vision, avoidance of major blood vessels and nerves, and safe, adequate spinal decompression.^10^ However, this approach also has significant disadvantages, including insufficient kyphosis correction, larger surgical trauma, and increased postoperative complications. In this study, we believe that the subscapularis transthoracic approach need to cut the musculi pectoralis major, latissimus dorsi, trapezius, serratus anterior muscle, rhomboid muscle, and vertical spine muscle. This approach also resulted in a longer surgical time and larger operation wound than the posterolateral approach. A higher incidence of postoperative shoulder movement disorder occurred due to the separation of loose connective tissue between the scapula and the chest wall (9.5% incidence in this study). Moreover, the kyphosis correction was limited because the spinous process, lamina, and other rear structures could not be removed via the anterior approach. In our study, kyphosis correction was greater in in group B (68.5%) than in group A (30.9%), which agrees with the conclusion of Pinglin et al.^18^ Therefore, posterior surgery is more appropriate for patients with obvious kyphosis.

Menard first employed the posterolateral approach for the treatment of upper thoracic tuberculosis; subsequently, many confirmed the safety and efficacy of this approach.^12^ The advantages of posterolateral approach include a single incision for the debridement, spinal decompression, bone graft and internal fixation; less operation trauma; low incidence of postoperative shoulder dysfunction; more reliable pedicle screw fixation; more effective kyphosis correction; and better recovery of physiological spine curvature. However, this approach also has some disadvantages, such as a longer fixed segment and the destruction of the structure of the rear normal spine. In this study, we found no significant difference in the recovery of neurological function between the 2 groups. We also found that the posterolateral approach can also be performed safely and effectively for spinal decompression.

Some experts have argued that the posterolateral approach combined with lesion debridement is unsafe because it may destroy the healthy posterior spinal column, leading to spinal instability.^19,20^ In our study, of the 22 patients with upper thoracic tuberculosis who were treated by the posterolateral approach, none exhibited failed internal fixation or failed fusion. Although the structure of the rear normal spine was destroyed via the posterolateral approach, the strength and long length of the segment pedicle fixation prevented any failure of the internal fixation or fusion, as evaluated during follow-up examinations. We believe that the posterolateral approach with resection of unilateral lamina, facet, transverse, and a small part of the ribs achieves effective postoperative fusion and does not cause upper thoracic instability. Possible reasons are the smaller mobility of the upper thoracic area relative to the cervical vertebra, lumbar vertebra and stronger capability of pedicle screw with good support, and anti-thoracic flexion, torsion. Intraoperative interbody fusion may also reestablish spinal stability.

In brief, the posterolateral approach is a feasible and effective method for the treatment of upper thoracic tuberculosis. In this study, it was superior to the subscapularis transthoracic approach. The posterolateral approach allowed for smaller surgical trauma and shorter operating time, and patients treated with this approach achieved remarkable kyphosis correction in their final follow-up evaluation. Due to the short follow-up times and low sample numbers in this study, there is a need for additional multicenter studies and studies with larger sample sizes to verify the clinical efficacy of this approach.

## CONCLUSION

Both surgical approaches improved patients’ Frankel scores and radiological results; however, there was no significant difference between the groups. This study provides evidence for the efficacy of both the subscapularis transthoracic approach and the posterolateral approach with debridement, bone graft fusion, and internal fixation for the surgical treatment of upper thoracic tuberculosis. The posterolateral approach offers a shorter operative time and better kyphosis correction; therefore, it may be a better surgical treatment for upper thoracic spinal tuberculosis.
